# Abnormal Movement Preparation in Task-Specific Focal Hand Dystonia 

**DOI:** 10.1371/journal.pone.0078234

**Published:** 2013-10-22

**Authors:** Jakob Jankowski, Sebastian Paus, Lukas Scheef, Malte Bewersdorff, Hans H. Schild, Thomas Klockgether, Henning Boecker

**Affiliations:** 1 FE Funktionelle Neurobildgebung, Radiologische Universitätsklinik, Universität Bonn, Bonn, Germany; 2 Neurologische Universitätsklinik, Universität Bonn, Bonn, Germany; 3 Radiologische Universitätsklinik, Universität Bonn, Bonn, Germany; 4 German Center for Neurodegenerative Diseases, Bonn, Germany; University of Iowa Carver College of Medicine, United States of America

## Abstract

Electrophysiological and behavioral studies in primary dystonia suggest abnormalities during movement preparation, but this crucial phase preceding movement onset has not yet been studied specifically with functional magnetic resonance imaging (fMRI). To identify abnormalities in brain activation during movement preparation, we used event-related fMRI to analyze behaviorally unimpaired sequential finger movements in 18 patients with task-specific focal hand dystonia (FHD) and 18 healthy subjects. Patients and controls executed self-initiated or externally cued prelearnt four-digit sequential movements using either right or left hands. In FHD patients, motor performance of the sequential finger task was not associated with task-related dystonic posturing and their activation levels during motor execution were highly comparable with controls. On the other hand reduced activation was observed during movement preparation in the FHD patients in left premotor cortex / precentral gyrus for all conditions, and for self-initiation additionally in supplementary motor area, left mid-insula and anterior putamen, independent of effector side. Findings argue for abnormalities of early stages of motor control in FHD, manifesting during movement preparation. Since deficits map to regions involved in the coding of motor programs, we propose that task-specific dystonia is characterized by abnormalities during recruitment of motor programs: these do not manifest at the behavioral level during simple automated movements, however, errors in motor programs of complex movements established by extensive practice (a core feature of FHD), trigger the inappropriate movement patterns observed in task-specific dystonia.

## Introduction

In primary dystonia, abnormal central nervous system (CNS) function has been documented in nearly every region that is relevant for motor control and sensorimotor processing [1,2]. Therefore, it has been argued that dystonia is a motor system or motor circuit disorder, rather than a disease of a particular motor region within the CNS. A number of mechanisms involved in the pathophysiology of dystonia have been identified recently [1,2], including deficient neuronal inhibition [3], and altered neuronal plasticity [4-6]. Furthermore, abnormalities of sensorimotor processing [7] and an abnormal amplification of the brain postural system [8] have been suggested to cause dystonia. Structural imaging techniques [9,10], and, in particular, functional neuroimaging like positron emission tomography (PET) and functional MRI (fMRI), have substantially contributed to our understanding of the disease. Abnormalities have been observed within various regions of the sensorimotor system, often related to specific sensorimotor functions [10,11]. However, functional imaging studies have yielded variable results, making it difficult to relate them to neurophysiological and cellbiological findings, and to integrate them into a conclusive pathophysiological model. Some of the possible reasons for these inconsistencies are different and sometimes mixed patient populations, relatively small patient numbers in some studies, use of different control groups, and use of variable motor tasks (for a more detailed discussion see 11-14. It has been shown, for instance, that abnormalities of brain activity in focal dystonia are correlated with task complexity [10,15-18], duration [19], and severity of symptoms [13,15,19]. 

So far, functional imaging studies in dystonia have paid little attention to abnormalities specifically related to distinct phases of movement, although it is known that the patterns of activated brain regions depend on the phase of the movement analyzed [20-23]. In fact, there is evidence from electrophysiological studies for disturbances prior to unimpaired [24-28] as well as dystonic movements [28-32], and fMRI of imagined movements in patients with focal hand dystonia (FHD) [33,34] indicate impairments of the motor control system that are not strictly associated with motor execution. However, to our knowledge, there is no fMRI study in focal dystonia, which explicitly analyzed movement preparation and motor execution in one setup, and thereby enabling control of task performance. In task-specific dystonia, symptoms may appear even upon the intention to perform a specific task, suggesting the generation of a disturbed motor strategy already before movement onset [2,35-37]. In line with this notion, an fMRI study by de Vries et al. [38] described abnormalities upon motor imagination as well as during clinically normal hand movements in patients with cervical dystonia. When patients with imagined grasping a pencil to either write with it or sharpen it in an fMRI study, Delnooz et al. found an abnormal activity in the dorsal premotor cortex for the "writing" condition [33]. Finally, in another fMRI study, Castrop et al. observed deficient cortical as well as subcortical activation in FHD patients during motor imagery of drawing simple geometric figures as compared to an observation task [34]. 

To gain a more comprehensive picture of the distribution of abnormal brain activity during movement preparation, defined as the phase between instruction and movement onset, we applied a previously established paradigm focusing on motor preparation with event-related whole-brain fMRI [22]. To preclude secondary effects caused by dystonic symptoms, a sequential finger task was used, which does not elicit dystonic posturing. Owing to the high variability of previous functional imaging findings in dystonia, it was important to ensure equal performance in matched, sufficiently large samples of patients and controls [10], and to avoid mixed patient populations. We therefore focused on FHD as task-specific focal dystonia. Studying both hands allowed for detection and characterization of effector independent abnormalities. Finally, to take into account observations of disturbed movement initiation in behavioral [39,40] and electrophysiological studies of dystonia [24,41], we compared self-initiated and externally cued movements.

## Materials and Methods

### Subjects

A prerequisite for inclusion into the study was that patients and healthy subjects were right-dominant in the Edinburgh Handedness Inventory and used only their right hand for writing (before and after manifestation of the disease). Patients were diagnosed with isolated idiopathic FHD according to medical history and clinical examination. Secondary dystonia was excluded by evaluation of disease course and MRI scans of the brain. Biochemical tests ruled out Wilson’s disease. Patients had never suffered from another neurological or psychiatric disorder, and had never taken neuroleptic medication. Only one potential patient was not be included in the fMRI study due to insufficient motor task performance upon behavioral testing. The final group of 18 patients was characterized by task-specific dystonia manifesting during handwriting (simple FHD), while in 12 patients, dystonia appeared also during other manual tasks (complex FHD), however not during the finger movement task as required for the study (see below). None of the patients presented with dystonia at rest or had dystonia in other body regions. For detailed clinical information, including the manual tasks eliciting dystonia in complex FHD patients, see Table 1.

**Table 1:** Subject data.

		**Controls**		**Patients**						
**Nr.**	**Gender**	**Age**	**EHI**	**Age**	**EHI**	**FHD**	**ADD**	**BFM**	**Duration**	**BoNT**
**1**	F	21.2	80	18.9	90	simple	0.81	2	11 year	20 weeks
**2**	F	49.4	45	49.1	80	complex	0.55	2	88 years	14 weeks
**3**	M	46.7	65	48.0	75	complex	0.51	3	77 years	2 years
**4**	M	34.6	80	35.5	40	complex	0.45	3	110 years	2 years
**5**	M	52.6	90	56.8	100	simple	0.80	1	66 years	1 year
**6**	M	28.6	75	26.3	85	simple	0.70	2	44 years	no treatment
**7**	M	52.5	55	49.1	20	complex	0.35	3	110 years	no treatment
**8**	M	44.4	45	41.0	15	complex	0.35	3	117 years	4 years
**9**	M	62.8	100	65.8	60	complex	0.70	2	330 years	10 years
**10**	M	57.0	50	57.6	95	complex	0.70	3	115 years	12 years
**11**	F	59.9	65	66.1	95	simple	0.65	2	223 years	13 weeks
**12**	M	58.1	35	57.9	15	complex	0.40	3	229 years	6 years
**13**	M	67.9	70	67.5	50	simple	0.75	2	334 years	4 years
**14**	M	54.1	85	61.8	50	simple	0.70	1	77 years	7 years
**15**	M	24.3	100	22.3	60	complex	0.60	2	11 year	no treatment
**16**	M	40.8	75	40.3	60	complex	0.60	2	33 years	no treatment
**17**	F	28.7	60	28.0	100	complex	0.70	2	113 years	no treatment
**18**	M	44.5	80	45.5	80	complex	0.60	2	110 years	12 weeks
**Average**		**46.0**	**69.7**	**46.5**	**65.0**	**n.a.**	**0.61**	**2.2**	**12.7 years**	**n.a.**
**SD**		15.5	28.7	13.8	18.8	n.a.	0.14	0.65	10.1 years	n.a.
		**Age**	**EHI**							
***P* Value^a^**		.92	.56							

EHI: Edinburgh Handedness Inventory; FHD: Focal Hand Dystonia; ADD: Arm-Dystonia-Disability Scale; BFM: Burke-Fahn-Marsden Scale; Duration: disease duration; BoNT: last botulinum neurotoxin treatment before fMRI; M: male; F: female; SD: standard deviation; n.a.: not applicable

a p-value of two-sided t-test of independent samples (unequal variance; Levene-test p < .05)

Average age of disease onset was 34 ± 11 years (range, 15 - 54 years), and average disease duration was 13 ± 10 years (range, 1 - 34 years). To assess the severity of FHD in each patient, we applied the Arm Dystonia Disability Scale [42], resulting in an average score of 61 ± 14 % (range, 35 - 81 %). Thirteen patients had been treated with botulinum toxin before, and those were studied at least three months after the last injection. Any drug effects had worn off at the time of the fMRI scan. No patient took any other medication for treatment of dystonia. None of the matched, healthy control subjects had ever suffered from any neurological or psychiatric disease (individual patient and control subject data are given in Table **1**).

### Protocol Approval and Patient Consents

The study was approved by the local Ethics Committee of the University Hospital, University of Bonn (Ethikkommission an der Medizinischen Fakultät der Rheinischen Friedrich-Wilhelms-Universität Bonn), according to national legislation and the Declaration of Helsinki. Patients and control subjects were enrolled after written informed consent.

### Task

**Figure 1:**
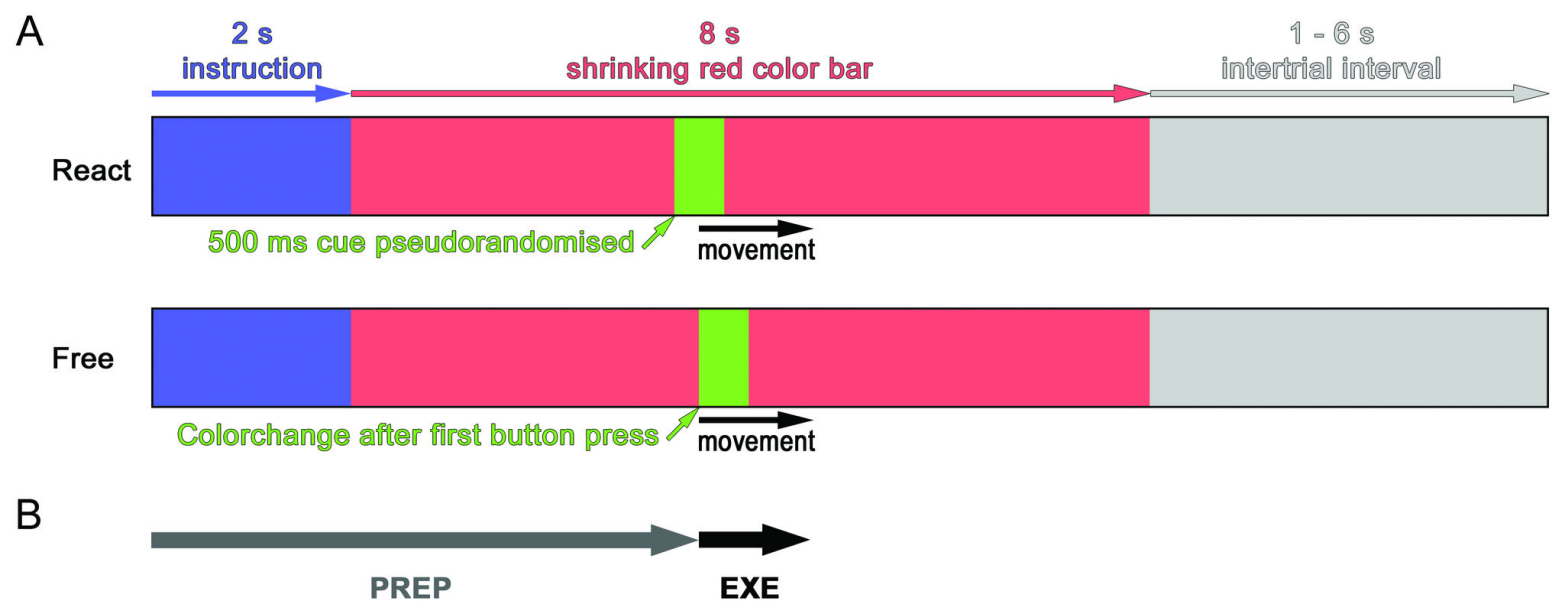
Experimental Paradigm. **A**: Experimental paradigm (trial). Black arrow: finger sequence. React-condition (upper bar): movement onset is delayed with respect to the cue (green). Free-condition (lower bar): movement onset triggers color change (green). **B**: Phases modeled for data analysis: preparation (PREP), execution (EXE).

The task has been previously applied in healthy subjects [22] and consisted of the following fixed sequence of four button presses: index, ring, middle, and little finger. For each individual trial, subjects were visually instructed which hand to use and about the mode of initiation: Either self-initiated (Free), where subjects could freely choose within a given time frame when to start the movement, or externally cued (React), where subjects had to start the movement following a (pseudo)randomized color change. Each trial consisted of the following elements (Figure **1 A**): 1) A visual instruction (2 s), specifying the condition (Free-L, Free-R, React-L or React-R). 2) A time frame (8 s), during which the movement had to be started, indicated by a horizontally oriented red bar, continuously shrinking from both sides until it disappeared at the center position. For the React-conditions, the time to start the movement was indicated by a 500 ms color change to green, which was programmed to appear at pseudo-randomized time points (mean 4.0 s, range 0.6 to 7.6 s after onset of the red bar). To avoid stereotypical movement initiation during the Free-conditions, subjects were instructed to vary the time points of initiation between individual trials. In this condition, the first button press induced a 500 ms color change of the bar from red to green. Before fMRI measurements, subjects were familiarized with the experimental procedure, and instructed to perform the predefined four-digit finger sequence at a comfortable pace, without requirement to respond to the cue as fast as possible, or to perform the sequence as fast as possible. Subsequently, subjects completed a 20 - 30 min training session at a personal computer using the same software application as used during scanning (see below). Before the experiment started, subjects rehearsed the sequence once more in the scanner for approx. 10 min so that they were able to perform it fluently at a comfortable and constant pace. For the "Free"-condition, they were instructed to vary the timepoint of movement initiation and to use the full range of the available time window (red color bar). Subjects practiced to randomly vary the time points for movement initiation and not to develop a specific strategy (like e.g. counting the trials, or performing "blocks" with successively similar time points of initiation). 

During fMRI, each of the resulting four conditions occurred 30 times, arranged in random order, and separated by variable intertrial intervals (mean duration 3.5 s, range 1.0 - 6.0 s). All motor responses during task execution were recorded using two MR-compatible optical response keypads (LUMItouch, Photon Control Inc., Burnaby, BC, Canada). 

### MRI Procedures

All stimuli were delivered using Presentation (Neurobehavioral Systems, Albany, CA, USA) and back-projected onto a semi-translucent screen outside the scanner. Subjects viewed the projection via a mirror mounted on the head coil. Functional MRI was performed on a 3.0T Achieva whole body MRI (Philips Medical Systems, Best, The Netherlands) using an 8 channel SENSE head coil (MRI-Devices) and a T2*-weighted gradient echo single-shot EPI sequence (TE / TR / flip angle = 35 ms / 2595 ms / 90°, sense factor 2, spatial resolution: 3.6 x 3.6 x 3.6 mm^3^). A total of 41 axial slices were recorded in interleaved ascending mode, which provided coverage of the entire brain (FOV: 230 x 230 x 147.6 mm^3^). In total, 800 volumes were acquired per fMRI-session. For all subjects, additional high-resolution structural images were acquired, using an MPRAGE-sequence (TE / TR / flip angle = 3.9 ms / 7.7 ms / 15°, spatial resolution: 1 mm^3^ isotropic, FOV: 256 x 256 x 180 mm^3^).

### Analysis of Behavior

Behavioral parameters were defined as follows: motor preparation (PREP) = time between appearance of the instruction and first button press; response time = time between cue (color switch) and first button press; motor execution (EXE) = time between first and last button press of finger sequence. Errors were defined as sequences of button presses other than the predefined sequence, wrong effector side, or button press outside the predefined time frame.

Data were statistically analyzed using a repeated measures analysis of variance (ANOVA), thereby taking into account that each subject was measured repeatedly under multiple conditions (within subject-factors). Statistics were performed in PASW 17.0 (Chicago, Illinois, USA). Between-subject factor "group" and within-subject factors "hand" and "condition", including possible interactions (hand x group, condition x group, and hand x condition x group) were analyzed for significant effects upon duration of motor preparation, motor execution, error rate, and response time, the latter being only available for the React-condition. Post hoc group comparisons were performed for behavioral parameters using a two-sided t-test of independent samples of equal or unequal variance (Levene-test p≥0.05 or p<0.05; Table **2**).

**Table 2:** Behavioral data.

**Parameter**	**Condition**	**Patients mean ± SD (range)**		**Controls mean ± SD (range)**		***P* Value** ^a^
Preparation phase [s]	Free-R	6.12 ± 0.65	(4.89 - 7.41)	6.20 ± 0.85	(4.79 - 7.82)	.74
	Free-L	6.21 ± 0.63	(4.56 - 7.20)	6.43 ± 0.88	(5.26 - 7.87)	.41
Response time [s]	React-R	0.44 ± 0.10	(0.28 - 0.66)	0.56 ± 0.22	(0.34 - 1.00)	.04^b^
	React-L	0.44 ± 0.10	(0.29 - 0.63)	0.57 ± 0.24	(0.34 - 1.09)	.04^b^
Movement [s]	Free-R	1.47 ± 0.49	(0.53 - 2.82)	1.64 ± 0.43	(0.73 - 2.77)	.27
	Free-L	1.53 ± 0.49	(0.58 - 2.76)	1.67 ± 0.44	(0.79 - 2.80)	.38
	React-R	1.39 ± 0.47	(0.43 - 2.60)	1.61 ± 0.41	(0.71 - 2.69)	.15
	React-L	1.45 ± 0.47	(0.49 - 2.66)	1.62 ± 0.40	(0.75 - 2.68)	.25
Errors per condition [n]	Free-R	2.0 ± 1.9	(0 - 6)	1.3 ± 1.2	(0 - 4)	.18
	Free-L	1.9 ± 2.9	(0 - 11)	1.5 ± 2.0	(0 - 7)	.64
	React-R	2.2 ± 1.6	(0 - 5)	1.9 ± 2.0	(0 - 8)	.72
	React-L	2.6 ± 2.6	(0 - 11)	1.8 ± 2.4	(0 - 9)	.33

^a^ two-sided t-test of independent samples of equal or ^b^ unequal variance (Levene-test p < .05)

### MRI Data Analysis

Pre-processing of the MRI data was performed in SPM5 (Wellcome Dept. of Imaging Neuroscience, London, UK). After slice time correction, all functional images were spatially realigned and unwarped to the first image to correct for head motion during the session. The realigned functional images were spatially normalized to the default EPI template in MNI space, as provided by SPM5. Subsequently, the data were interpolated to a voxel size of 3.0 x 3.0 x 3.0 mm^3^ and smoothed using an isotropic Gaussian kernel of 8 x 8 x 8 mm^3^. For the analysis depicted in Figure **S1**, no smoothing was applied. 

At the first level, data were analyzed on a voxel-by-voxel basis using the principles of the general linear model [43], including a 128s high pass filter, and modeling PREP and EXE as behaviorally defined epochs by specifying onset and duration (see behavioral parameters and Figure **1B**). Modeling durations was important to account for the duration range of the PREP-phase (2.6 s to 10.0 s). The design matrix consisted of the event types PREP and EXE for each of the four movement conditions (Free-R, Free-L, React-R and React-L), resulting in a total of 8 regressors of interest, which were modeled using the canonical hemodynamic response function in SPM5. Error events and movement parameters were included as regressors of no interest.

The following functional contrasts were analyzed:

1 Motor preparation of self-initiated (Free-R_PREP_, Free-L_PREP_) and externally cued conditions (React-R_PREP_, React-L_PREP_) compared to baseline.
2 Motor execution of self-initiated (Free-R_EXE_, Free-L_EXE_) and externally cued conditions (React-R_EXE_, React-L_EXE_) compared to baseline.
3 Differential brain activity comparing motor preparation of self-initiated and externally cued conditions (Free-R_PREP_ > React-R_PREP_, React-R_PREP_ > Free-R_PREP_).

For within-group analyses, contrast images from first level were entered into second-level random effects analyses, and considered significant at a height threshold of p<0.001 (FDR-corrected) and an extent threshold of k=10 voxels. For between-group comparisons, two-sample t-tests of unequal variance were performed, and considered significant at an uncorrected height threshold of p<0.001 and an extent threshold of k=10 voxels. Anatomical localization of the activation peaks was determined using the Talairach Client software (Version 2.4.2, http://www.talairach.org/; [44,45]) and the SPM Anatomy Toolbox 1.5 [46]).

## Results

### Behavioral data

Behavioral data are given in Table **2**. Statistical analysis revealed highly comparable task performance, differing between groups only in average response times (p=0.048, F=4.15; shorter in FHD patients), but not for group x hands. Within groups, movement duration was significantly different with respect to hand (p=0.002, F=10.7) and condition (p<0.001, F=26.3). The average duration of the PREP-phases was very similar for all conditions in both groups. However, it was significantly different between conditions (p=0.014; F=6.6) for methodological reasons, as the duration of the PREP-phase was determined by the stimulation program in the React-condition, whereas it was determined by the subjects themselves in the Free-condition. 

### Functional MRI data

#### Within group analysis

**Figure 2:**
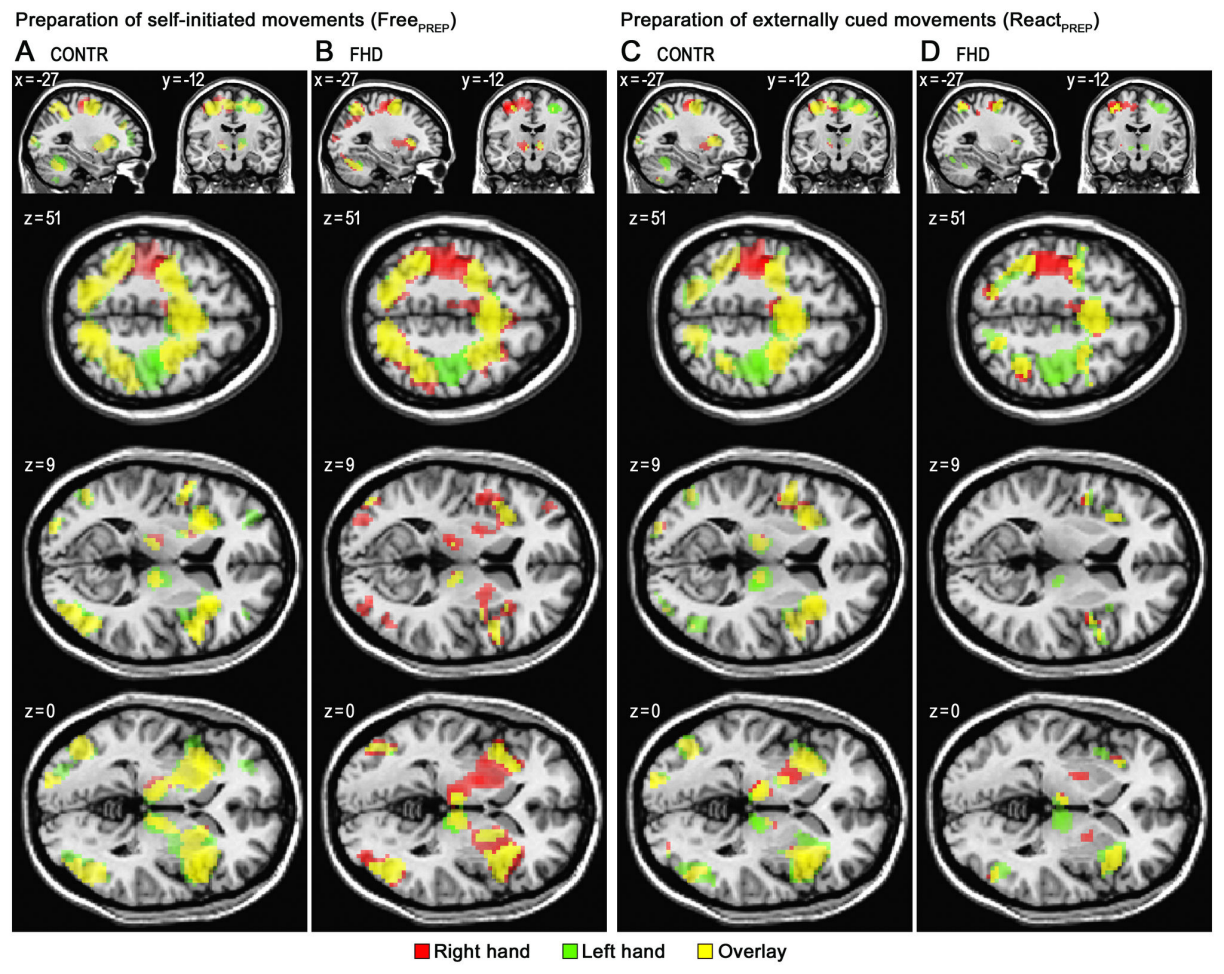
Within-group Activity during Preparation of Movements. Within-group activity during preparation of movements in healthy control subjects (CONTR; A and C) and focal hand dystonia patients (FHD; B and D). Activation during motor preparation (PREP) of self-initiated (Free; A and B) and externally cued (React; **C** and **D**) conditions. Voxels surpassing a height threshold of p < 0.001 (FDR-corrected) and an extent threshold (cluster size) of k=10 are superimposed on the MNI-T1-template of SPM5, and color coded for right hand (red), left hand (green) and common activations (overlay; yellow). Coordinates shown (x, y, z) are in MNI-space.

Both, healthy controls and FHD patients showed motor preparation-related activation in premotor, superior and inferior parietal cortical regions, insula, and subcortically in basal ganglia and thalamus, extending into the midbrain (Figure **2**). During motor execution, activations were shifted towards more lateralized, posterior motor executive regions, as described previously [22,23]. At the descriptive level, comparison of motor preparation-related activity revealed an asymmetry for FHD patients not observed in controls, with lower activity within the left basal ganglia.

#### Between group comparisons

Statistical comparison of patients and controls revealed decreased activations in pre-motor networks for FHD patients during motor preparation (PREP, see below). No differences were detected during motor execution (EXE) at the same level of significance (p<0.001 uncorrected, k=10 voxels). 

**Figure 3:**
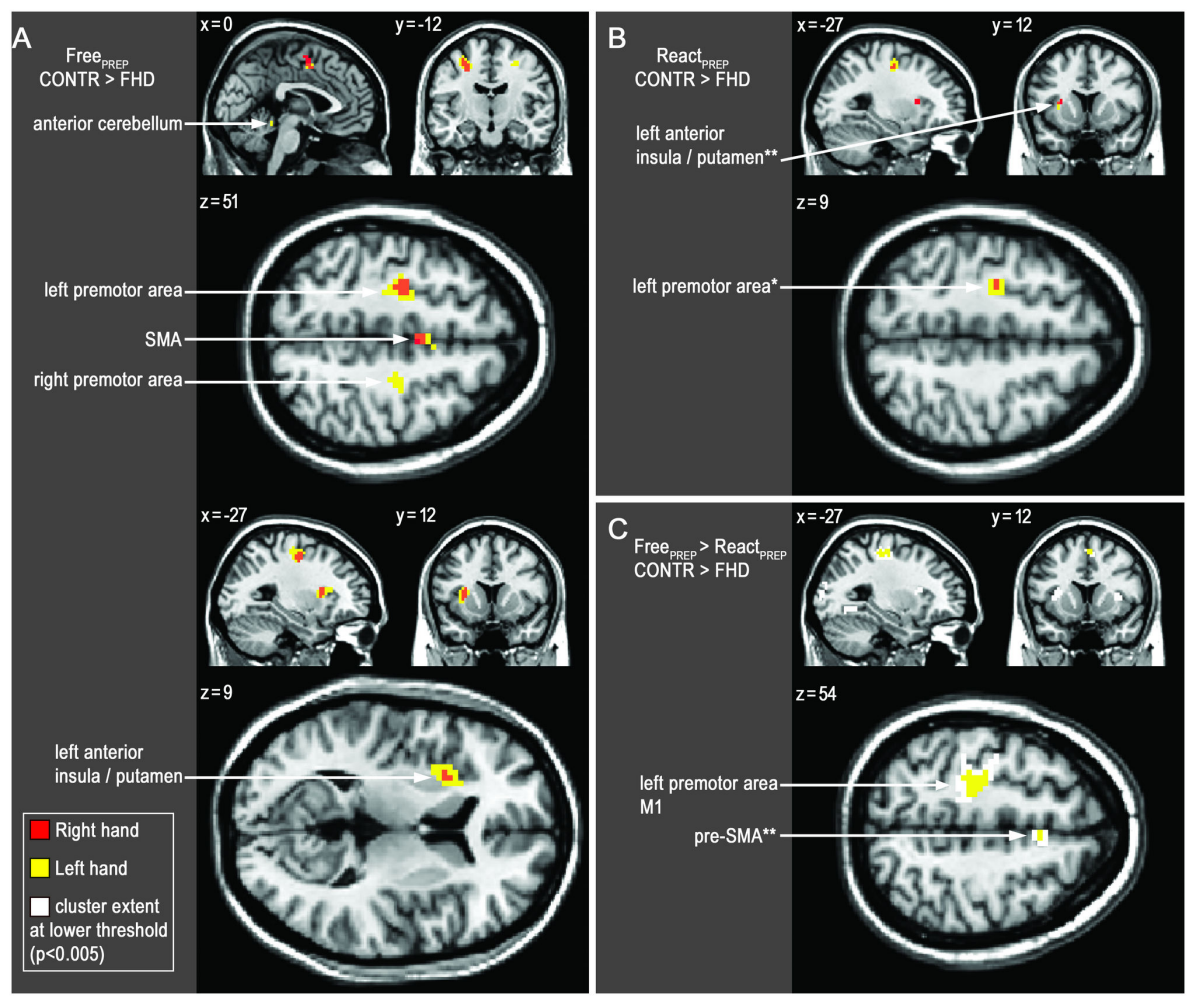
Between-group Activation Changes during Preparation of Movements. Decreased activity in patients (FHD) compared to controls (CONTR) during motor preparation (PREP). p<.001 (uncorrected), extent threshold 10 voxel. Right hand (red), left hand (yellow). **A**: Self-initiated **B**: Externally cued **C**: Reduced self-initiation-related activity (white: p<0.005). Note effector independent distributions. Coordinates shown (x, y, z) are in MNI-space.

During movement preparation of externally cued movements significant group differences were seen for left hand (React-L_PREP_), where underactivity in FHD patients was detected within the left premotor area (PMA; BA 6) bordering M1 (Figure **3B**, Table **3C**). No significant group differences were seen for the affected right hand (React-R_PREP_), however, when lowering the extent threshold for React-R_PREP_ to k=5 voxels, underactivity was detected in the same region, and additionally within a cluster encompassing mid-insula and anterior putamen (Figure **3B**, Table **3B**). 

**Table 3:** Functional imaging data.

**A**	**Free-R_PREP_ (CONTR > DYS)**	**BA**	**K**	**T**	**Z**	**x**	**y**	**z**
*Mid.*	*Medial Frontal Gyrus (SMA)*	*6*	*19*	*3.95*	*3.56*	*0*	*0*	*51*
*Left*	*Premotor Cortex / Precentral Gyrus*	*6*	*21*	*4.26*	*3.79*	*-27*	*-12*	*51*
	*Middle Insula (Anterior Putamen)*	*14-16*	*14*	*3.95*	*3.56*	*-27*	*15*	*12*
**B**	**Free-L_PREP_ (CONTR > DYS)**							
*Mid.*	*Medial Frontal Gyrus (SMA)*	*6*	*12*	*4.05*	*3.63*	*0*	*0*	*51*
	Cerebellum (Anterior Vermis)		12	3.80	3.45	6	-42	-15
				3.55	3.25	-3	-42	-15
*Left*	*Middle Insula (Anterior Putamen) ^b^*	*14-16*	*66*	*5.31*	*4.50*	*-27*	*12*	*9*
	*Premotor Cortex / Precentral Gyrus ^b^*	*6*	*73*	*5.11*	*4.37*	*-27*	*-12*	*51*
Right	Premotor Cortex / Precentral Gyrus	6	13	4.58	4.01	27	-15	51
	Posterior Insula	13	19	4.55	4.00	42	-24	-3
	Insula^a^	14-16	31	4.36	3.86	51	6	0
				4.05	3.63	42	-3	0
**C**	**React-R_PREP_ (CONTR > DYS)**							
*Left*	*Middle Insula (Anterior Putamen)*	*14-16*	*5*	*3.86*	*3.49*	*-27*	*12*	*9*
	*Premotor Cortex / Precentral Gyrus*	*6*	*5*	*3.65*	*3.33*	*-27*	*-12*	*48*
Right	Hippocampus	20	5	3.75	3.41	33	-21	-9
**D**	**React-L_PREP_ (CONTR > DYS)**							
*Left*	*Precentral Gyrus ^a^*	*6*	*45*	*4.76*	*4.14*	*-27*	*-12*	*51*
**E**	**Free-L_PREP_>React-L_PREP_ (CONTR>DYS)**							
Left	Precentral Gyrus ^b^	6	53	4.45	3.92	-24	-21	54
		6/4p		3.81	3.45	-18	-27	51
		4p/3a		3.64	3.32	-33	-15	48
Mid.	Superior Frontal Gyrus (SMA)	6		3.64	3.32	6	12	54

^a^ p_(uncorrected)_ cluster level < .05; ^b^ p_(corrected)_ cluster level < .05 height threshold: p < .001 uncorrected, cluster size threshold k = 10 voxel (except for **C**)

BA: Brodmann area; K: cluster size (voxel); T: T-value; Z: Z-score; x, y, z: coordinates in MNI-space [mm]. *Italics* indicate identical locations of clusters for two or more conditions

During motor preparation of self-initiated movements (Free_PREP_) underactivity became more obvious within exactly the same regions (left PMA, mid-insula, anterior putamen; Figure **3A**, Table **3A**), and additionally within supplementary motor area (SMA). For the left hand (Free-L_PREP_), these effects were more pronounced than for the right hand, showing additional deficits within right PMA, posterior parts of the right insula and anterior cerebellar vermis (Figure **3A**, Table **3B**). To further resolve the cluster encompassing left putamen and insula, between group analysis of unsmoothed data was performed, showing a local maximum located within the mid-insula as well as an adjacent (sub-threshold) maximum within the anterior putamen (Figure **S1**).

Comparing differential contrasts (self-initiated vs. externally cued) between FHD patients and controls during left-sided performance (Free-L_PREP_>React-L_PREP_) revealed relatively decreased activity in left PMA (BA6) extending into primary motor cortex (M1, BA4), and within the SMA (Figure **3C** **** Table **3E**). 

## Discussion

### Impaired motor programs in task-specific dystonia

The outstanding task-specificity of some primary focal dystonias has led to the hypothesis of impaired motor programs [29,35]. Such a "motor program" can be thought of as neuronal assemblies and defined interactions of brain areas, representing detailed feed-forward information on how to perform a skilled movement, such as writing or playing an instrument (for critical reviews see 47,48). These motor programs are established during practice, and are constantly updated and refined during repeated performance by modification of neuronal interactions and excitability [49]. 

The observation, that in task-specific dystonia symptoms may appear even upon the sole intention to perform a task, point to abnormalities during movement preparation where recruitment of a specific motor program occurs. Based on our findings, we would like to propose that such motor programs are altered in task-specific dystonia, which manifests already during recruitment of motor programs and even in the absence of dystonic symptoms. In the following, we will discuss our findings with respect to the various phases of motor control, will specifically consider the different brain regions found to be involved, and will relate these findings to current pathophysiological concepts of dystonia. 

### Movement preparation

To our knowledge, this is the first fMRI study specifically characterizing movement preparation and motor execution in one setup in task-specific dystonia prior to movements not disturbed by dystonic posturing, and thereby enabling control of task performance. In FHD patients, reduced brain activity was observed within pre-motor regions during movement preparation of both, the clinically affected and unaffected hand, in spite of a behaviorally normal motor performance. A similar distribution of deficits of brain activity in FHD patients has been described in an fMRI study of imagined drawing (a paradigm targeting motor control independent of motor execution), namely within the left primary sensorimotor cortex, left premotor cortex, bilateral putamen and thalamus [34], however a relation of these deficits to the effector side and an assessment of motor output was not possible in the motor imagery task. In our study, hypoactivity was largely confined to almost identical brain regions independent of the executing hand, located within the left hemisphere, which was suggested to play a critical role "in instantiating sensorimotor engrams that support control of skilled movements irrespective of hand" [50]. This argues for abnormalities of an established motor program in task-specific dystonia. On the cortical level, the neural correlates of such abnormalities could be a loss of inhibitory function, as observed intracortically prior to movements in focal hand dystonia [3], or during suppression of motor programs in a Go/NoGo trial in musicians’ dystonia [51].

### Motor execution

No statistically significant group differences were detected during motor execution of the bahaviorally unimpaired prelearnt sequential finger task. At first sight, this seems to be at odds with previous functional imaging studies, observing disturbed brain activity during movement [10,11]. However, since the analysis of motor execution revealed highly specific and significant activation patterns within each of the matched and comparatively large groups, we assume that during unimpaired task performance brain activity in patients does not differ substantially from healthy subjects. This notion is supported by electrophysiological studies, observing deficits before, but not during motor execution of simple movements [24,26,28]. Furthermore, the failure to detect abnormal brain activity during motor execution in our study, which seems to be in contrast to previous functional neuroimaging studies, can be explained - to a large extent - by methodological differences. Some of the abnormalities observed in other studies are obviously related to unequal task performance, and the frequently intentional induction of dystonic symptoms [19,52,53]. Moreover, studies using block-design fMRI or PET are unable to distinguish abnormalities of movement preparation and motor execution. In line with our findings, when using tasks, which did not induce dystonic symptoms, predominantly decreased activity was observed in patients [10,13,17,18,54]. Furthermore, there are findings of hypoactivation in patients with cervical dystonia [38] and FHD [34] during motor imagery tasks, which can be assumed to emphasize primarily aspects of motor preparation and have the advantage that resulting activation levels are not affected by abnormal motor output (similar to the rationale of studying unimpaired motor tasks pursued in the current study). Nevertheless, due to the absence of on-line EMG recordings during fMRI we have to acknowledge that we cannot exclude very subtle abnormalities during motor execution that are not detectable upon visual control. Moreover, it is possible that extended task duration would have revealed such functional abnormalities [19].

### PMA/M1 and SMA

According to a recent meta-analysis [55], the locations of the effector-side independent cortical deficits observed here map to the borders between left PMA and M1, and pre-SMA and SMA, regions involved in movement preparation. In line with impaired motor programs in FHD, these specific locations seem to be involved in the encoding of trained movements: They were found to be part of a bilateral network for the control of complex finger movements independent of the executing hand [56]. During writing, they were activated independently of the executing limb (hand or foot), prompting the authors to conclude that the "functional representation of a highly trained movement ... is coded in neural assemblies that are part of the anatomical representation of the limb with which it is usually performed" [57]. In professional piano players, considerably reduced activity has been observed within these regions during finger tapping [58], supposedly reflecting lower inhibitory neuronal activity during execution of excessively trained movements. Training might enhance performance of highly complex tasks by selectively reducing inhibition, as observed in healthy professional musicians when compared to non musicians [49,59]. However, in task-specific dystonia, these physiological adjustments seem to be disturbed, interfering with - rather than sharpening - movements, since surround inhibition was decreased even further in musicians dystonia [60]. Supporting this notion, in an fMRI study, FHD patients exhibited an abnormally low cortical activity during complex, behaviorally unimpaired movements [18].

Although showing a partly identical distribution within the brain, the effects observed in the current study appeared to be more pronounced during motor preparation of movements executed by the left hand. A potential explanation of this difference is that the deficits become more apparent when increasing task demand by the requirement to use the non-dominant hand, in line with findings of Wu et al., who observed a correlation of activation deficits with the complexity of the task in dystonia patients [17]. However, it is important to point out that a direct statistical comparison of left and right hand movement preparation did not reveal any statistically significant differences. Furthermore, we included handedness as covariate in the model, which did not reveal any effects of handedness on the dystonia-related activation patterns (data not shown).

### Mid-insula

Although frequently found to be activated during somatosensory and motor tasks, little attention has been paid to the role of the insular cortex in motor control [61]. In dystonia, several studies demonstrate abnormal activation of the insula [13,15,52,62], however, only some of them comment on its potential function [13,15,62]. More generally, the insula has been ascribed an integrative role, linking information from diverse functional systems [63]. The mid-insula is connected to somatosensory and motor cortices [64], and might be involved in sensorimotor processing [63]. In fact, hypoactivity observed in FHD patients (MNI -27, 12, 9) colocalizes with the maximum for the category "motion" (including all motor tasks; MNI -29, 12, 9) of a recent meta-analysis on insular function [63]. This underactivity might therefore reflect a deficit when matching motor programs to the requirements of the specific situational context in which the movement shall be performed, potentially triggering an overshooting (compensatory) activity leading to dystonic symptoms during highly complex and trained movements like writing. 

### Anterior putamen

In dystonia, deficits of the indirect pathway and consecutive lack of inhibitory basal ganglia output has been held responsible for overexcitability observed in dystonia [7,14,65]. On the other hand, impairment of the direct pathway has been proposed, which is thought to facilitate execution of specific learned motor tasks [66-68]. The cluster of decreased activity encompassing the insula also extended into the left anterior putamen and within-group analysis revealed a distinctly asymmetrical distribution of activity in FHD patients, with relatively less activity within the left putamen, arguing for a functional deficit in this region (Figure **2** and Figure **S1**). In line with this, a recent fMRI study observed deficient basal ganglia-premotor activation during motor imagery, leading the authors to propose impaired basal ganglia inhibition and focusing during the selection of motor programs in dystonia [34]. However, since the involved pathways (indirect and direct pathway) and their functional interconnections are still far from being fully resolved, at present, it is difficult to define the precise role of the putamen for motor preparation in the context of task-specific dystonia (for discussion, see e. g. 69,70). For instance, another potential cause for a seemingly reduced activity could be a locally increased baseline function in dystonia patients. In fact, Blood et al. demonstrated a persisting elevation of basal ganglia activity after performance of finger-tapping tasks [71]. However, if such differences of baseline activity were the origin of the differences observed here, we would expect to detect them also during motor execution, and rather within similar locations as described by Blood et al. (i.e. pallidum and posterior putamen), which both are not the case. Finally, the induction of a persisting elevation of baseline activity might require a prolonged motor activity, like the continuous 60 s bilateral motor task applied in the cited study, in contrast to the short four finger sequence performed in our paradigm. 

### Mode of Initiation

The deficits observed in this experiment depended on the mode of movement initiation: Whereas impairments within the left lateral PMA were seen for all conditions, hypoactivity of the SMA became obvious only for self-initiated tasks. Directly comparing motor preparation of the self-initiated with the externally cued condition revealed lower activity in PMA, M1 and pre-SMA in the FHD patients as compared to the controls,. This is in line with current concepts of SMA function [72] and neuroimaging work showing activity in the SMA to be particularly relevant for preparation of self-initiated movements [20,21,73]. In dystonia, the association of abnormalities within the SMA with movement complexity [17] and its modulation by botulinum neurotoxin treatment (BoNT) [52], argue for a more variable, task dependent abnormality of the SMA in this condition.

### Pathophysiological interpretation of task-specific dystonia

Integrating these findings with current neurophysiological concepts, our data are congruent with a pathophysiological model of task-specific dystonia centered around errors in the organization of established motor programs [29,35]: In task-specific dystonia, altered neuronal plasticity has been described [4,6,74], which is thought to result in the establishment of faulty motor programs, particularly affecting complex movements requiring extensive practice and repeated performance [49,60]. Notably, abnormal sensorimotor processing [7,37,75] might interfere with the correct establishment and recruitment of motor programs. Correlates of these impairments in task-specific dystonia might be deficits of neuronal inhibition [3], manifesting as reduced activity of inhibitory interneurons [14,41] during recruitment of motor programs, which is consistent with abnormalities in the EEG prior to movement onset [28-30,32] and also abnormal BOLD-signals during motor imagery tasks [33,34]. Hypoactivity (i.e., decreased BOLD-signals) during movement preparation, as observed here, fits perfectly well into this concept, since the BOLD-signal predominantly reflects activity related to local neuronal input and processing, including inhibitory activity [76,77]. Furthermore, increased output activity of projection neurons could even be masked by this hypoactivity [77], in line with the absence of detectable differences during motor execution. However, in dystonia inducing tasks or during prolonged movements, hyperexcitation and massively increased local processing due to sensory feedback caused by dystonic symptoms, will eventually dominate the picture obtained by BOLD-fMRI. Therefore changes of the ratio of local inhibitory activity and output activity might influence direction (increase or decrease) of the signal measured, generating variable results when studying different groups of subjects. For example, Lim et al. [78] observed increased EEG activity (late contingent negative variation, CNV) prior to movements in pianists with focal dystonia when comparing them to healthy professional pianists, whereas others [30,79] observed decreased CNV in FHD patients in comparison to healthy subjects. A possible explanation for such differences might be, that the extent of intracortical inhibition and excitability is altered (physiologically) in professional musicians and (pathologically) in patients with task-specific dystonia [49,60], compared to normal subjects. Furthermore, the pattern of dystonia related activation changes observed is highly dependent on a number of variables defined by the study design, like the movement phase, the type of dystonia analyzed, the affected body part, the experimental task (degree of symptoms induced, duration and complexity), only to name a few. It is therefore crucial to control for these variables and for equal task performance in patient and control groups. In fact, much of the variability observed in functional neuroimaging studies in focal dystonias can be explained by such methodological differences, and a number of apparent discrepancies can be resolved when taking into account these differences [10,11]. 

Although the concept of neuronal assemblies constituting specific "motor programs" is still a matter of debate [48], our line of argumentation based on faulty motor programs is not only a basis for explaining the task-specificity of dystonic symptoms, but may also help to understand several other conspicuous clinical characteristics of focal dystonia: For instance, after switching handwriting to the other hand, with increasing practice the initially unaffected hand can become clinically affected; in a cohort of 14 patients learning to write with their unaffected limb, simple motor difficulties were described in four patients and dystonic difficulties in one patient in their previously unaffected hand [80]. This could be explained by the recruitment of an existing faulty motor program (e.g. for writing). However, when starting to learn to use the non-dominant hand for writing, only limb-independent components of the existing motor program can be used, requiring increased (sensory) feedback-control, counteracting the occurrence of dystonic symptoms. In the further course of relearning and with sustained practice, reliance on sensory feedback-control is continuously reduced, until, after limb-specific "refinement" of the motor program, the threshold for manifestation of dystonic symptoms is reached. Furthermore, the concept of limb-independent motor representations might help to explain why the dystonia usually does not spread to other body regions. Differences in the individual thresholds for the manifestation of the errors in such motor programs may also explain the large interindividual range of when and whether a patient with hand dystonia will develop dystonic symptoms in the previously unaffected hand [80]. Exerting a sensory-trick ("geste antagoniste") [81] might in a similar way activate feedback-mechanisms and thereby counteract dystonic symptoms. Importantly, these unconscious feedback-mechanisms have to be distinguished from attempts to consciously overcome dystonic symptoms. In conclusion, as there were no significant differences in abnormalities during the motor preparation phase for the dystonic and the non-dystonic limbs, our data are compatible with a concept of limb-independent abnormalities of motor representations. 

A hypothesis by Blood [8] suggests that dystonia is an abnormal amplification of the brain postural system. She proposes that the action of the postural system is required for the dynamical fine-tuning of the movement, to finally enable the correct execution within a given setting. With an increasing number of individual movements which must be coordinated and fine-tuned in order to perform a task (e. g. writing or playing an instrument), the degree of automatization necessary to achieve maximum speed and precision increases. Integrating our findings into this hypothesis, increasing the complexity of the task might increase the likelihood that errors within the underlying motor program trigger an over-activation of the movement-controlling postural signals, resulting in "excessive dynamic postures".

### Limitations

A major limitation of fMRI per se is the inability to distinguish between increases in BOLD-signal caused by input activity, local integration or output activity, as well as inhibitory and excitatory activity [76,77]. For example, a high local activity consisting of inhibitory and excitatory activity can cause a strong BOLD-signal increase, without resulting in any efferent output activity. Vice versa, a decreased inhibitory activity can cause an excitatory output in spite of an unchanged or even decreased regional cerebral blood flow [82]. For the interpretation of fMRI-data, it is therefore indispensable to integrate these with information obtained from other modalities. Since it is very well in agreement with neurophysiological findings [3], we propose that the hypoactivities observed here are caused by impaired (local or afferent) inhibitory activity. However, an alternative explanation would be a less focused distribution of brain activity within specific regions of the motor network in FHD patients. As has been shown in schizophrenia patients, group averaging may underestimate regional activation because of increased heterogeneity of the location of activation peaks within certain brain areas [83]. Accordingly, inter-individually more variable locations of activity would result in a lower signal increase on the within-group level, manifesting as a relative decrease in group comparisons. Abnormal patterns of cortical [84] as well as subcortical [85] brain activity within sensorimotor regions have been observed in patients with dystonia, and it has been shown in animal models that repetition and overuse can cause degradation of receptive fields [86]. In animals, invasive techniques are available, allowing detection and neurophysiological analysis of changes within small anatomical units [87,88]. However, owing to the technical limitations of fMRI and the whole-brain approach, we are unable to distinguish, whether hypoactive (inhibitory) neurons are diffusely distributed or instead confined to units such as motor cortex columns, as proposed by Guehl et al. [88]. For the same reason we can not answer the question, whether the abnormalities observed in FHD patients are caused by a reduced local activity (e. g., impairments of intracortical inhibition [3]), or whether they are a result of abnormal (e. g., subcortical) afferent inputs, in line with an abnormal surround inhibition [67], or both. Due to the limited temporal resolution of functional neuroimaging, we cannot determine the initial anatomical origin of the disturbed brain activity during movement preparation as observed here. Nevertheless, by identifying deficits specifically related to the phase prior to movement onset, we are able to distinguish them from secondary disturbances caused by abnormal movement execution. This is indeed further supported by our paradigm, which did not induce overt dystonic symptoms. 

A methodological limitation of the current study is that no EMG recordings were performed, as minor dystonic movements might have stayed undetected. However, as we observed BOLD underactivity during the motor preparation phase in the dystonia group, it is rather unlikely that undetected minor dystonic movements have biased our interpretation of the imaging data, as these would be expected to cause BOLD increases. Furthermore, the absence of differences between patients and controls during the motor execution phase argues for the absence of relevant dystonic symptoms. Nevertheless, EMG should be included in future imaging works investigating dystonia-inducing tasks. 

### Outlook

As the reported fMRI abnormalities were by definition not strictly related to dystonic symptoms, it would be an interesting extension of this work to study also the motor preparation phase prior to dystonic symptoms. A population where this aspect could be investigated in a meaningful manner would be musician’s dystonia. Here, a highly specific dystonia-inducing movement, i.e. piano playing, could be compared with a very similar movement not inducing dystonia, i.e. sequential finger movements which involve the same muscle groups and comparable movement patterns. Such a feature is not applicable in the investigated population of FHD patients, where e.g. a sequential finger movement would have to be compared with handwriting, thus, substantially limiting comparability due to grossly different movement types. Another interesting approach for future research would be to study motor preparation longitudinally prior to disease onset until full clinical manifestation, as this would allow identifying whether abnormalities in motor preparation occur as constituent effects early in the disease process, or as secondary long-term effects induced by dystonic symptomatology. 

## Conclusions

By separately analyzing movement preparation and motor execution for the affected and unaffected hand, we observe a pattern of hypoactive brain regions (premotor cortex / precentral gyrus for all conditions, and for self-initiation additionally in supplementary motor area, left mid-insula and anterior putamen) in FHD patients, which support a pathophysiological model of task-specific dystonia, in which abnormal brain activity prior to movement onset is a consequence of faulty motor programs. Relating these findings to existing pathophysiological concepts, establishment of these faulty motor programs in task-specific dystonias may be caused by disturbed sensorimotor processing and altered neuronal plasticity, and manifest as deficient neuronal inhibition. 

## Supporting Information

Figure S1 **Distribution of hypoactivity in focal hand dystonia (FHD) patients within the insula and the basal ganglia using unsmoothed data.** Between-group differences of activity (decreased activity in patients compared to healthy subjects (CONTR > FHD; blue)) are compared to the distribution of within-group activity of healthy subjects (CONTR; red) during preparation of self-initiated movements using the left hand (Free-L_PREP_). Between-group analysis (blue; voxels surpassing a height threshold of p < 0.001, uncorrected) is superimposed on the within-group analysis (red; voxels surpassing a height threshold of p < 0.001, FDR-corrected) and on the MNI-T1-template of SPM5. Coordinates shown (x, y, z) are in MNI-space. Within-group analysis (CONTR; red) reveals clearly distinguishable clusters in the anterior putamen and the mid insula. Between-group analysis (CONTR > FHD; blue) shows a distinct hypoactivity within the mid insula, but also to a lesser extent hypoactivity located within the anterior putamen (right column).
(TIF)
