# Supplementary figures and images for: Abnormal Movement Preparation in Task-Specific Focal Hand Dystonia
Source: PLoS One. 2013 Oct 22;8(10):e78234. doi: 10.1371/journal.pone.0078234 (PMC3805688; doi:10.1371/journal.pone.0078234)

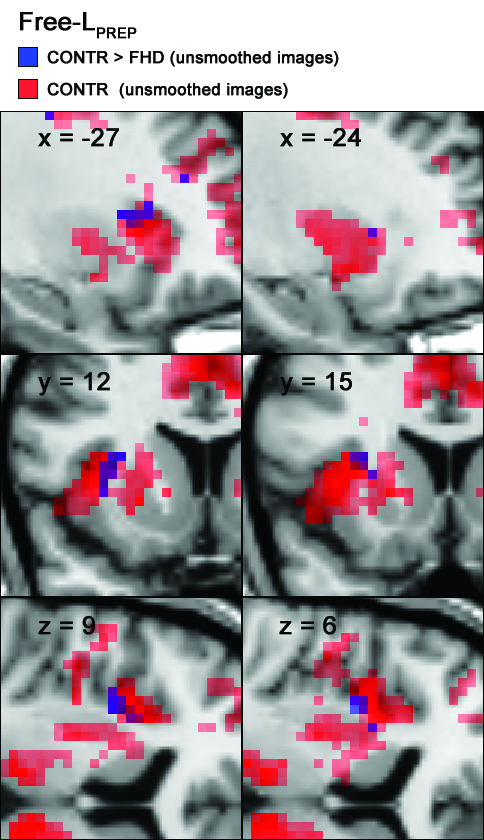

Supplement: Figure S1 — Distribution of hypoactivity in focal hand dystonia (FHD) patients within the insula and the basal ganglia using unsmoothed data. Between-group differences of activity (decreased activity in patients compared to healthy subjects (CONTR > FHD; blue)) are compared to the distribution of within-group activity of healthy subjects (CONTR; red) during preparation of self-initiated movements using the left hand (Free-LPREP). Between-group analysis (blue; voxels surpassing a height threshold of p < 0.001, uncorrected) is superimposed on the within-group analysis (red; voxels surpassing a height threshold of p < 0.001, FDR-corrected) and on the MNI-T1-template of SPM5. Coordinates shown (x, y, z) are in MNI-space. Within-group analysis (CONTR; red) reveals clearly distinguishable clusters in the anterior putamen and the mid insula. Between-group analysis (CONTR > FHD; blue) shows a distinct hypoactivity within the mid insula, but also to a lesser extent hypoactivity located within the anterior putamen (right column). (TIF) [file pone.0078234.s001.tif]
